# Suspected Sildenafil-Induced Immune Hemolytic Anemia Precipitating Acute Kidney Injury Requiring Hemodialysis

**DOI:** 10.7759/cureus.89690

**Published:** 2025-08-09

**Authors:** Bryan Koithara, Shashikala Sangle, Supriya Barsode, Nitin Gadkari, Aditya Jadhav

**Affiliations:** 1 Internal Medicine, Bharati Vidyapeeth (Deemed to be University) Medical College and Hospital, Pune, IND

**Keywords:** acute kidney injury, direct antiglobulin test, drug-induced immune hemolytic anemia, hemodialysis, sildenafil citrate

## Abstract

Drug-induced immune hemolytic anemia (DIIHA) is a rare secondary cause of autoimmune hemolytic anemia (AIHA), more frequently associated with drugs such as cephalosporins, penicillin, non-steroidal anti-inflammatory drugs (NSAIDs), and certain chemotherapeutic agents. The condition is often underdiagnosed due to marked variability in antibody type and affinity, resulting in inconsistent serological findings. Such delays increase the risk of hemolytic crisis, which may result in target end-organ failure or death.

We report the case of a 26-year-old man with no prior comorbidities. He presented to our emergency department with an acute history of passing dark-colored urine, followed by reduced urine output for two days. Preliminary laboratory investigations revealed severe anemia, elevated lactate dehydrogenase, and indirect hyperbilirubinemia, suggesting a hemolytic pathology and a strongly positive direct antiglobulin test (DAT), which was consistent with a diagnosis of immune hemolytic anemia. This presentation was complicated by severe acute kidney injury (AKI). The patient's renal parameters progressively improved after initiating renal replacement and corticosteroid therapy. Despite extensive investigations, no underlying trigger for immune-mediated hemolysis could be identified. It was at this point that the patient disclosed recent recreational use of sildenafil citrate before symptom onset.

Utilizing the Naranjo adverse drug reaction probability scale, we were able to isolate sildenafil as a probable trigger for immune-mediated hemolysis. The present case raises the possibility of sildenafil as a previously unreported trigger for drug-induced immune hemolytic anemia (DIIHA).

## Introduction

Autoimmune hemolytic anemia (AIHA) is a relatively rare, potentially life-threatening condition caused by IgG-mediated destruction of erythrocytes. It may be idiopathic or occur secondary to infections, autoimmune diseases, malignancies, or drugs. While more than 130 drugs have been suspected to induce immune hemolytic anemia, cephalosporins, non-steroidal anti-inflammatory drugs (NSAIDs), and chemotherapeutic agents are frequently implicated [1]. Drug-induced immune hemolytic anemia (DIIHA) is recognized as a secondary cause of AIHA, and every suspected case of DIIHA warrants a systematic search as per the recommendations of the first international consensus on AIHA diagnosis and treatment [2]. While the incidence of AIHA was estimated to be between one and three per 100,000 per year based on nationwide population-based studies, DIIHA is a far rarer occurrence with an estimated incidence of one per million population [3-5]. Sildenafil, a widely used phosphodiesterase-5 inhibitor for erectile dysfunction and pulmonary artery hypertension (PAH), has not previously been reported to cause immune-mediated hemolysis in humans. Experimental data suggest that sildenafil can destabilize red blood cell membranes and increase susceptibility to lysis in in vitro human studies [6]. In this case, the etiology of AIHA remained unclear until the patient retrospectively disclosed sildenafil use. Given the potential for rapid deterioration, early recognition of DIIHA and prompt discontinuation of the offending agent are essential to mitigate morbidity and prevent renal or hematological complications.

## Case presentation

A 26-year-old man with no known comorbidities presented to our emergency department with a two-day history of passing dark-colored urine, followed by a progressive reduction in urine output, culminating in anuria at the time of admission. He also gives a history of vague groin pain accompanied by generalized weakness and fatigue. He is a strict vegetarian, denies alcohol, tobacco, or drug abuse, and has no significant family history of hematological or renal disease. There are no complaints of fever, night sweats, weight loss, or respiratory complaints. He denies any recent strenuous or prolonged physical activity, regular medication use, and previous blood transfusion. His past medical history was unremarkable for such a presentation.

On initial evaluation, he is conscious, cooperative, and well-oriented. Vital signs show a blood pressure of 130/70 mmHg, a heart rate of 102 beats per minute, a respiratory rate of 16 cycles per minute, and an oxygen saturation of 97% on room air. A general physical examination reveals a well-built man who is pale but lacking any signs of icterus, cyanosis, finger clubbing, lymphadenopathy, or pedal edema. Cardiovascular, respiratory, and neurological examinations are unremarkable and non-contributory. Abdominal examination did not suggest any organomegaly.

Baseline investigations

Renal function tests revealed severe acute kidney injury (AKI). Serum myoglobin levels were undetectable, and serum creatine phosphokinase (CPK) was within normal limits. The patient was anuric by this time with a 24-hour urine output of 80 mL despite adequate intravenous fluid. A urine analysis showed elevated levels of urobilinogen and a positive dipstick test for protein and hemoglobin, but was free from red blood cells (RBCs) and casts. Severe hemolytic anemia precipitating AKI associated with elevated serum ferritin and low serum haptoglobin levels prompted us to screen for possible causes of intravascular hemolysis. However, tests to estimate glucose-6-phosphate dehydrogenase (G6PD) levels, paroxysmal nocturnal hemoglobinuria (PNH) by flow cytometry (FCM), rapid malaria test (RMT), dengue serology, and serological testing for hepatitis B surface antigen (HBsAg), anti-hepatitis C virus (HCV) antibody, and human immunodeficiency virus (HIV) returned negative or normal. Blood and urine cultures were sterile. Chest X-ray was unremarkable, but an abdominal ultrasound revealed borderline splenomegaly (12.2 cms), increased renal cortical echogenicity bilaterally, but with normal renal size and maintenance of corticomedullary differentiation.

**Figure 1:**
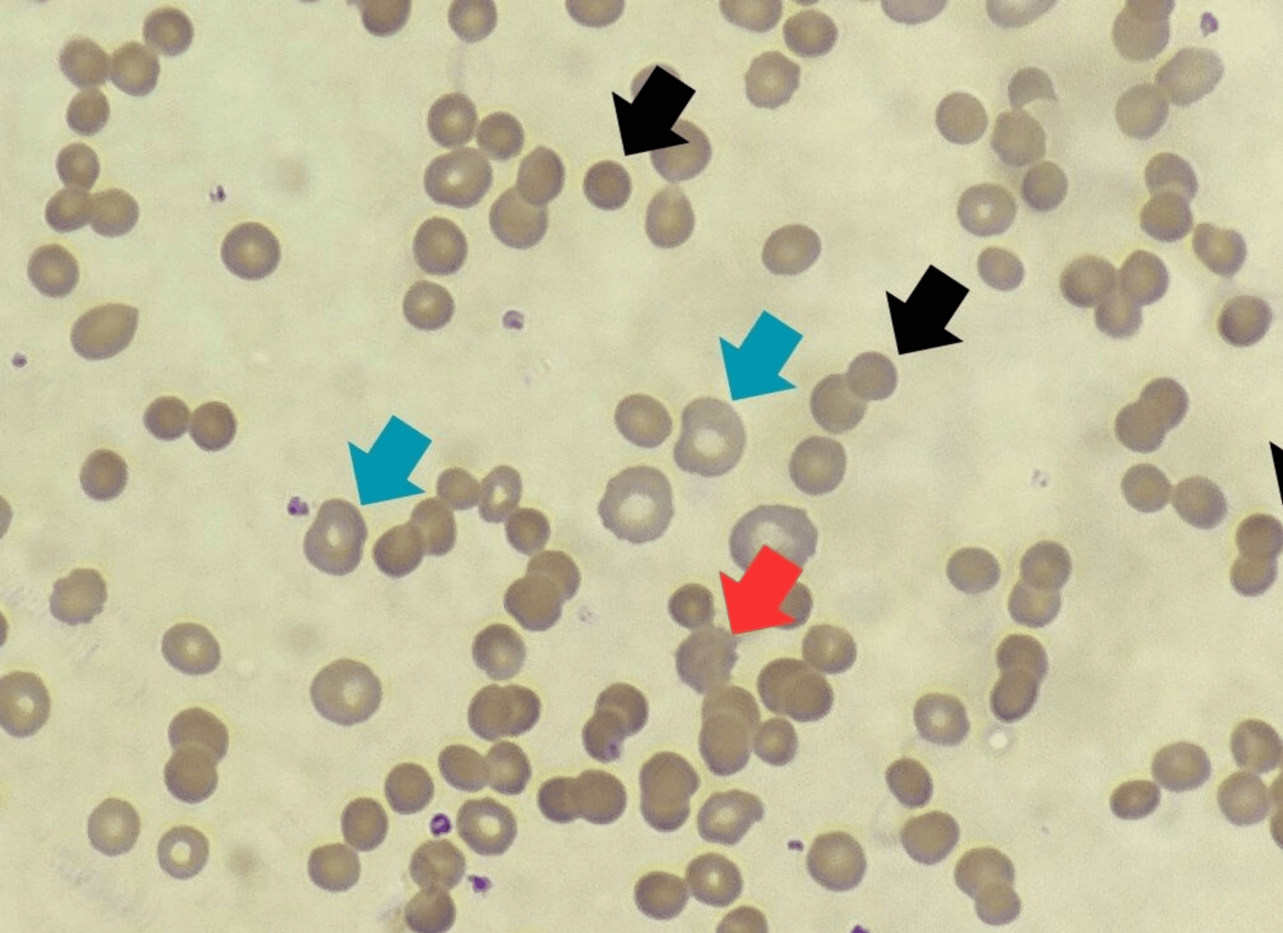
Photomicrograph of H&E-stained peripheral blood smear (100× magnification) showing microspherocytes (black arrow), macrocytes (blue arrow), and fragmented RBCs (red arrow) H&E: hematoxylin and eosin, RBCs: red blood cells

Further blood work revealed severe normocytic normochromic anemia, markedly elevated lactate dehydrogenase (1,535 U/L), indirect hyperbilirubinemia (2.2 mg/dL), and a reticulocyte count of 13%. Red cell indices showed adequate serum iron levels and adequate iron stores. Polyspecific and monospecific direct antiglobulin test (DAT) was strongly positive for both anti-IgG and anti-C3d, and the peripheral smear demonstrated microspherocytes, macrocytes, and fragmented RBCs without schistocytes (Figure 1).

**Figure 2:**
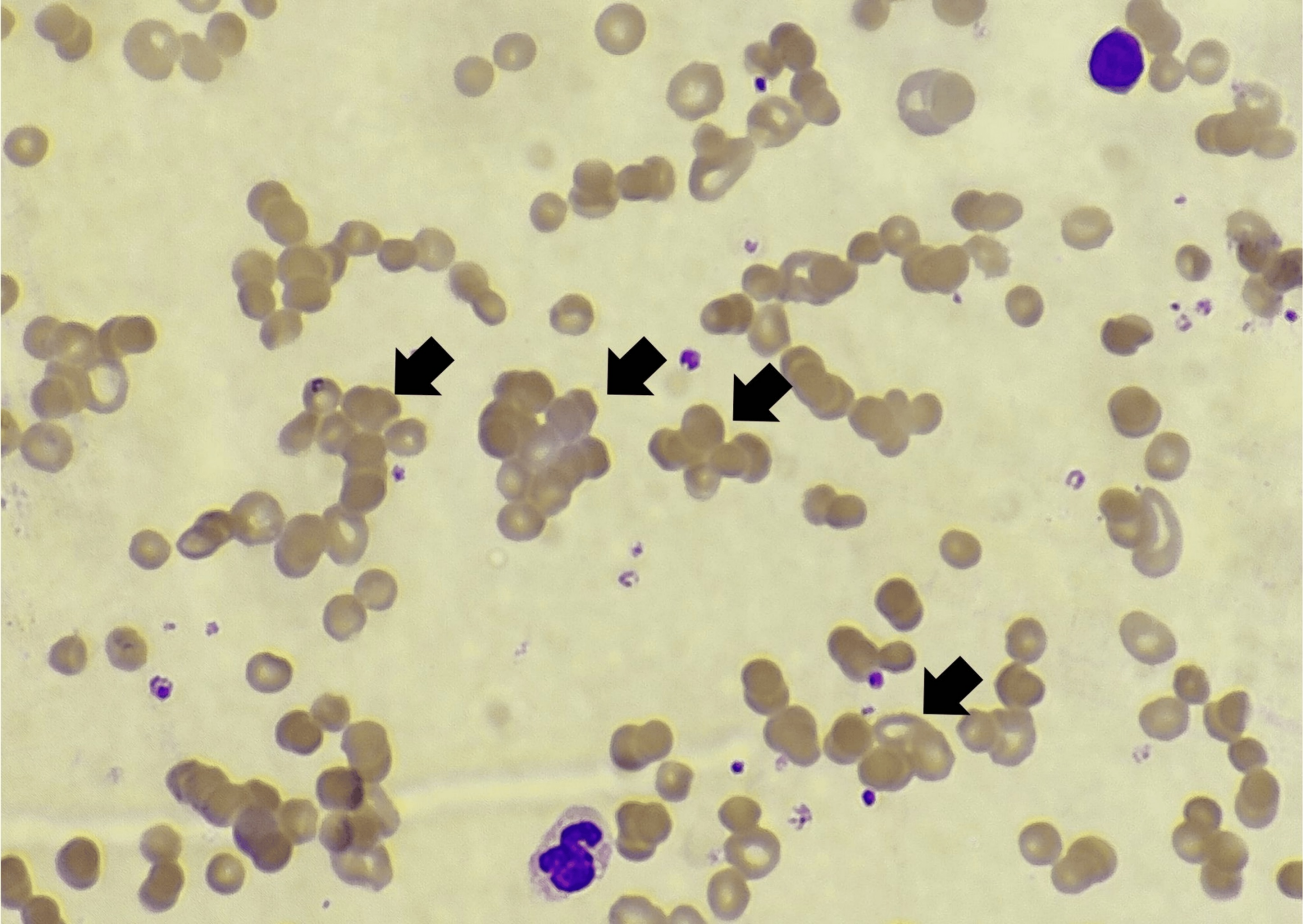
Photomicrograph of H&E-stained peripheral blood smear (100× magnification) showing extensive RBC autoagglutination (black arrow) H&E: hematoxylin and eosin, RBC: red blood cell

Notably, widespread RBC autoagglutination was observed (Figure 2).

A strongly positive direct antiglobulin test (DAT) allowed us to contemplate an immune-mediated mechanism. However, this diagnosis was initially challenged as tests for infectious triggers, such as dengue illness, malaria, viral hepatitis, and HIV, as well as autoimmune triggers, were all negative. A strongly matched RhD-positive B blood grouping further exacerbated this diagnostic conundrum. Autoantibodies encountered in warm AIHA are notorious for causing discrepancies during cross-matching. It was at this point during his hospitalization that the patient disclosed recent recreational use of sildenafil. While the exact dosage was uncertain, he had admitted to consuming at least six tablets of sildenafil citrate, the dose of each being 100 mg, amounting to a total dose of 600 mg, over a period of three days before symptom onset. A detailed summary of the laboratory and radiological findings is presented in Table 1.

**Table 1:** Outline of laboratory and radiological investigations Hb: hemoglobin, g: grams, dL: deciliter RBC: red blood cell, TLC: total leucocyte count, PLT: platelet, MCV: mean corpuscular volume, fL: femtoliters, MCH: mean corpuscular hemoglobin, pg: picograms, MCHC: mean corpuscular hemoglobin concentration, RDW: red cell distribution width, μg: microgram, ng: nanogram, UIBC: unsaturated iron-binding capacity, TIBC: total iron binding capacity, TSAT: transferrin saturation, LDH: lactate dehydrogenase, INR: international normalized ratio, AST: aspartate transaminase, ALT: alanine transaminase, ALP: alkaline phosphatase, CRP: C-reactive protein, G6PD: glucose-6-phosphate dehydrogenase, U: units, PNH: paroxysmal nocturnal hemoglobinuria, FCM: flow cytometry. CPK: creatine phosphokinase, CAT: column agglutination test, IFA: indirect immunofluorescence assay, ELISA: enzyme-linked immunosorbent assay, DAT: direct anti-globulin test, IAT: indirect anti-globulin test, HIV: human immunodeficiency virus, HBsAg: hepatitis B surface antigen, HCV: hepatitis C virus, RMT: rapid malaria test

Test	Result	Normal value
Hematological tests		
Blood grouping by CAT		
Anti A	Negative	-
Anti B	Positive	-
Anti D	Positive	-
Hemogram		
Hb (g/dL)	6.2	13-17
RBC count (million/mm^3^)	1.96	4.5-5.5
TLC (/mm^3^)	10,100	4,000-11,000
PLT count (/mm^3^)	234,000	150,000-450,000
Red cell indices		
Hematocrit (%)	18.7	40-50
MCV (fL)	95.3	83-101
MCH (pg)	34.8	27-32
MCHC (g/dL)	36.5	31.5-34.5
RDW (%)	15.2	11.6-14.0
Iron panel		
Serum iron (μg/dL)	123	65-175 (males)
Ferritin (ng/mL)	1,148.95	21.81-278.66 (males)
UIBC (μg/dL)	89	65-240 (males)
TIBC (ug/dL)	212	250-450
TSAT (%)	58.01	20-50
Markers of hemolysis		
Serum LDH (U/L)	1,535	125-220
Serum haptoglobin (mg/dL)	<14	30-200
Retic count (%)	13.0	0.2-5.0
Red cell proliferation index (%)	2.4	0.5-2.5
Indirect bilirubin (mg/dL)	2.5	0.2-0.7
Coagulation profile		
Prothrombin time (seconds)	10.6	10.1-14.0
INR	0.89	0.8-1.1
Activated partial thromboplastin time (seconds)	25.4	24-36
Immune hemolytic anemia tests		
Immunology		
ANA by IFA	Negative	-
Anti-dsDNA IgG by ELISA	Negative	-
Complement 3 (C_3_) (mg/dL)	88.47	90-180
Complement 4 (C_4_) (mg/dL)	27.60	10-40
Markers of intravascular hemolysis		
G6PD (quantitative) (U/g of Hb)	21	6.7-18.7
PNH panel by FCM	Negative	-
Antiglobulin test		
DAT by gel CAT		
Polyspecific DAT (IgG + C_3_d)	Positive (2+) at 37° Celsius, negative at 4° Celsius	
Monospecific DAT (IgG)	Positive (2+) at 37° Celsius, negative at 4° Celsius	
Monospecific DAT (C_3_d)	Negative at 4°and 37° Celsius	
IAT by gel CAT		
Polyspecific DAT (IgG + C_3_d)	Negative at 4° and 37° Celsius	
Monospecific DAT (IgG)	Negative at 4° and 37° Celsius	
Monospecific DAT (C_3_d)	Negative at 4° and 37° Celsius	
Microbiological tests		
Blood culture	Sterile	
Urine culture	Sterile	
HIV	Non-reactive	
HBsAg	Non-reactive	
Anti-HCV	Non-reactive	
RMT	Negative	
Dengue serology (IgM and IgG)	Negative	
Renal tests		
Urinalysis (sample: spot urine)		
pH	5.5	5-7.5
Specific gravity	1.020	1.000-1.030
Pus cells (per high power field)	3-4	0-5
Hb (dipstick)	+++	Absent
Urobilinogen	Elevated	-
No RBCs, eosinophils, or cast cells are present.		
Urinalysis (sample: 24-hour urine)		
24-Hour urine volume (mL)	80	800-1,200
Kidney function tests		
Serum creatinine (mg/dL)	8.42	0.55-1.02
Serum urea (mg/dL)	110	14.9-40.0
Liver function tests		
Total protein (g/dL)	5.3	6.4-8.7
Albumin (g/dL)	3.6	3.5-5.2
Globulin (g/dL)	1.7	2.3-3.2
AST (U/L)	44	<35
ALT (U/L)	34	<45
Serum ALP (U/L)	68	40-150
Total bilirubin (mg/dL)	3.3	0.3-1.2
Direct bilirubin (mg/dL)	0.8	0-0.5
Inflammatory markers		
CRP (mg/L)	3.4	<5
Miscellaneous tests		
Vitamin B_12 _(pg/mL)	<148	187-883
Myoglobin (mg/dL)	0	0-0.003
CPK (U/L)	51	39-308
Radiological investigations		
Chest X-ray	Normal study	
Abdominal ultrasound	Bilateral raised renal cortical echogenicity and splenomegaly (12.2 cms)	

To summarize, while a strongly positive DAT allowed us to confirm immune-mediated hemolytic anemia, the patient initially presented with features suggesting intravascular hemolysis, further bolstered by the detection of elevated levels of urobilinogen and hemoglobin in urine, while free of RBCs. Further evaluation for causes of intravascular hemolysis were non-contributory. Simultaneously, peripheral blood smear findings of microspherocytes, macrocytes, and fragmented RBCs aligned more consistently with an extravascular hemolytic pathology. Thus, we hypothesize a possible mixed intravascular and extravascular process occurring in our patient.

Treatment

**Figure 3:**
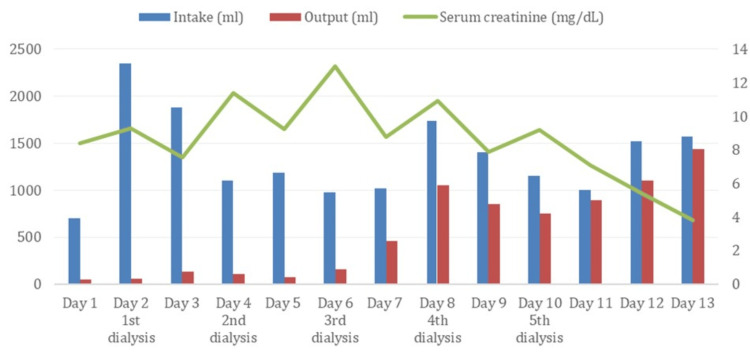
Fluid intake-output charting and creatinine trends throughout hospitalization The primary y-axis (far left) represents fluid intake-output in mL. The blue bar plots total fluid intake, while the red bar plots total fluid output. The secondary y-axis (far right) represents serum creatinine levels in mg/dL. The green line plots serum creatinine levels. The x-axis represents the day of hospitalization and dialysis sessions.

On confirmation of AIHA, the patient was started on intravenous methylprednisolone (500 mg once daily for three days) [7]. This was followed by high-dose oral prednisolone at 80 mg once daily due to persistent autoagglutination on peripheral smear and only modest hematological recovery. Urine output remained minimal despite fluid resuscitation and diuretic therapy. His serum creatinine rose to 10.5 mg/dL, indicating worsening AKI necessitating initiation of renal replacement therapy. Dialysis was initiated via a right internal jugular venous catheter for temporary access. Concurrently, he underwent five sessions of hemodialysis over the next 10 days and was transfused with two pints of packed RBCs. Blood transfusions were well tolerated without incident. The patient's intake-output charting and serum creatinine trend throughout hospitalization are summarized in Figure 3.

Outcome and follow-up

The patient's hemolysis parameters gradually improved throughout hospitalization. A repeat DAT turned negative by day 5 of oral steroids, accompanied by complete resolution of autoagglutination from the peripheral smear. His hemoglobin stabilized at approximately 10.2 g/dL.

Renal function showed partial recovery, and his serum creatinine stabilized at 3.8 mg/dL at the time of discharge. He was discharged in stable condition on oral vitamin B12 and folic acid supplements and oral prednisolone (80 mg/day) with a tapering plan. The patient was also counselled on strict avoidance of sildenafil and potential future risks of hemolytic relapse.

On his first follow-up at two weeks, the patient was healthy and asymptomatic. His hemoglobin had risen to 11.8 g/dL, and serum creatinine was 1.4 mg/dL. Oral prednisolone was tapered by 20 mg every week thereafter until a dose of 20 mg once daily was achieved by week 5. This was followed by a slow taper over the next four weeks and was finally omitted. At 10 weeks of follow-up, his serum creatinine had returned to baseline, and his hemoglobin was holding steady at 13.8 g/dL.

## Discussion

This case highlights a potential previously undocumented adverse reaction to sildenafil citrate: DIIHA complicated by AKI requiring renal replacement therapy.

The antibodies involved in DIIHA are broadly categorized into two types. The first type are drug-dependent antibodies that either react in vitro with drug-coated RBCs or when added to RBCs in the presence of the unbound drug in solution. These drug-dependent antibodies bind to red blood cells through various mechanisms, leading to complement activation or immune sensitization, and ultimately hemolysis via complement cascade activation or macrophage-mediated clearance. The second type is the drug-independent type, which involves RBC autoantibodies rather than antibodies against the drug itself.

**Figure 4:**
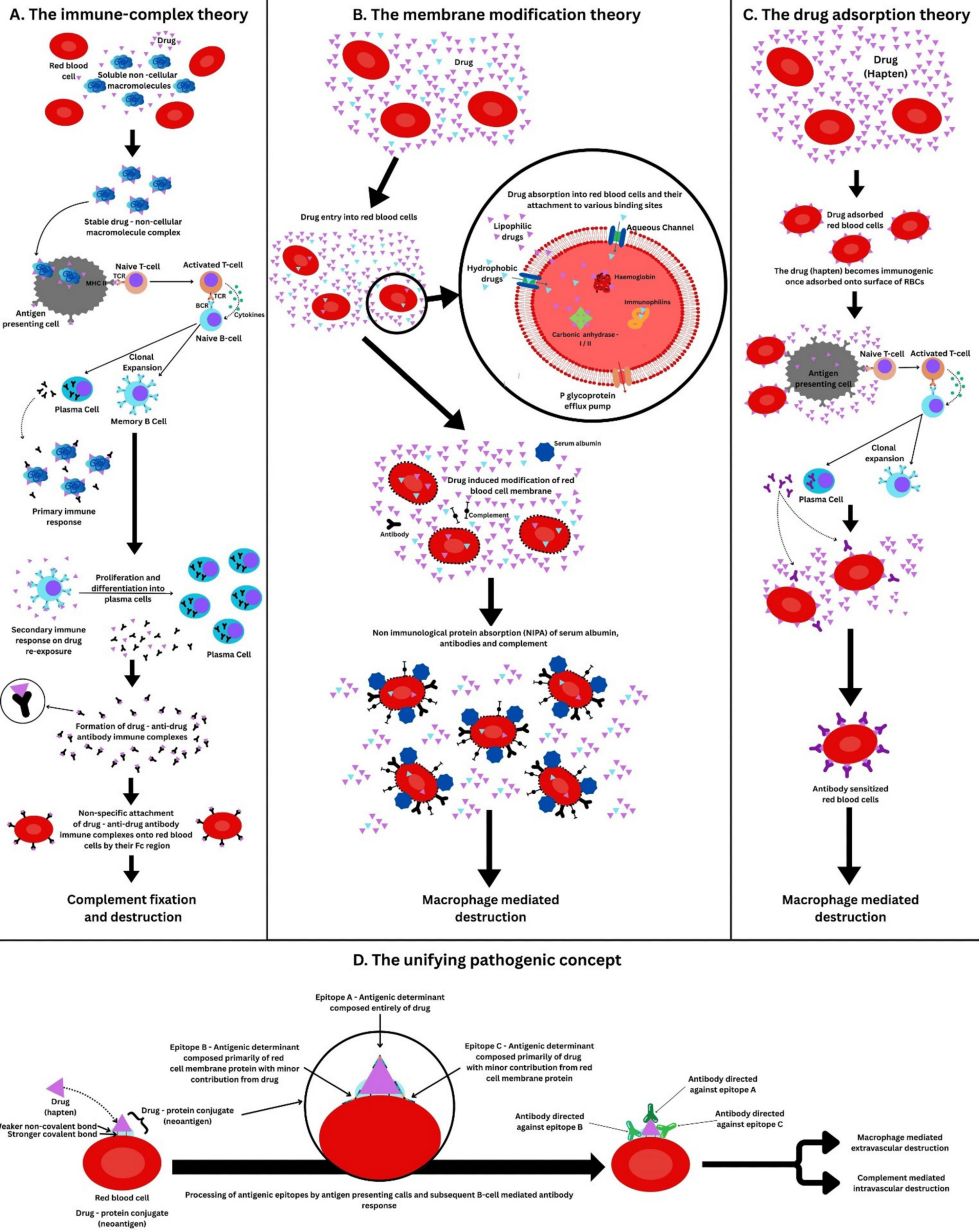
Proposed mechanisms for DIIHA A: The proposed mechanism of the immune complex theory. B: The proposed mechanism of the membrane modification theory. C: The proposed mechanism of the drug adsorption theory. D: The proposed mechanism of the unifying pathogenic concept, incorporating elements from the previous theories. DIIHA: drug-induced immune hemolytic anemia, NIPA: non-immunological protein absorption, TCR: T-cell receptor, BCR: B-cell receptor

Several mechanisms have been proposed to explain DIIHA [8]. The first, "immune complex" theory, suggested that patients form antibodies against stable complexes of the drug with soluble macromolecules, which then fix and activate complement on re-exposure. The second, "drug adsorption" theory, described how drugs like penicillin can adsorb onto the surface of RBCs in high doses, binding with anti-drug IgG antibodies and marking RBCs for destruction by macrophages. A third "membrane modification" theory involves non-immunological protein absorption (NIPA), in which the drug alters the RBC membrane, leading to the passive absorption of serum proteins, including antibodies and complement. A fourth "unifying pathogenic concept" combining these ideas was introduced. According to this theory, the drug binds with RBC membrane proteins by stronger covalent or weaker non-covalent bonding, forming drug-protein conjugates that act as neoantigens. This model explains how both drug-dependent and independent mechanisms might coexist, with epitopes varying in their drug and RBC contributions. Thus, multiple antibody populations may simultaneously contribute to DIIHA. Figure 4 illustrates these four mechanisms.

In the present case, despite the patient's laboratory results aligning more consistently with an intravascular hemolytic picture, the presence of borderline splenomegaly, accompanied by a peripheral blood smear devoid of schistocytes and bite cells but showing microspherocytes, suggested a parallel extravascular hemolytic pathology. Additionally, the occurrence of AKI necessitating the initiation of renal replacement therapy cannot be ignored. Hemolysis-induced AKI is believed to occur due to hemoglobin and other heme-proteins mediated direct tubular injury, renal vasoconstriction, and urinary cast formation [9]. Hemolysis precipitating AKI is frequently encountered with intravascular hemolysis, wherein free hemoglobin accumulates in plasma after saturating serum haptoglobin stores, thus allowing it to be filtered across the glomerular filtration barrier and subsequently endocytosed by proximal tubular cells. This endocytosed hemoglobin is then metabolized by heme-oxygenase, allowing for the accumulation of free heme and its sequestration in tubular cells by ferritin, producing a cytotoxic effect [10]. This corroborated with the urinalysis findings of our present case, which showed a positive dipstick for both hemoglobin and elevated urobilinogen levels. Thus, we hypothesize combined intravascular and extravascular hemolysis in our patient. These findings are consistent with existing literature describing DIIHA manifesting as intravascular or extravascular hemolysis depending on the extent of complement activation [11-13].

Sildenafil citrate is a phosphodiesterase-5 inhibitor that is widely prescribed for treating erectile dysfunction and pulmonary artery hypertension (PAH). Current guidelines recommend a maximum dose of 100 mg daily [14]. The dose our patient had taken was well above this recommended ceiling (600 mg over a three-day period) and hence warrants a discussion on whether DIIHA in our patient was a consequence of a dose-related toxicity versus an idiosyncratic reaction. Dose-related toxicity typically manifests as fever, headache, flushing, dyspepsia, nasal congestion, myalgia, nausea, dizziness, fixed-drug eruptions, and epistaxis [15]. Rarer manifestations include visual disturbances and ototoxicity [16,17]. However, our patient did not manifest or give a history of any of these complaints on presentation. Furthermore, single doses of 800 mg have been administered to normal volunteers, and all that was observed was an increased risk of the usual dose-related adverse events [15]. Sildenafil, however, has not been previously reported as a cause of DIIHA in humans. These observations support the hypothesis that DIIHA secondary to sildenafil ingestion was more likely an idiosyncratic reaction. Although in vitro studies have shown sildenafil-induced changes in RBC membrane integrity, these findings do not confirm an immune-mediated hemolytic mechanism and should be interpreted cautiously [6]. Additionally, while the occurrence of sildenafil-induced acute tubular necrosis (ATN) precipitating AKI has been documented previously, the presentation of oliguric AKI in the present case suggests hemolysis-induced AKI as the predominant pathogenic mechanism for renal injury [18].

Utilizing the Naranjo adverse drug reaction probability scale, the implicated drug had a score of 5: 2 points for the occurrence of an adverse event after the suspected drug was consumed, 1 point for improvement of adverse event after the suspected drug was discontinued, and another 2 points for the absence of alternative causes that could have produced a similar reaction [19]. The components of the Naranjo adverse drug reaction probability scale and their interpretation are summarized in Table 2.

**Table 2:** Naranjo adverse drug reaction probability scale Interpretation of the Naranjo adverse drug reaction probability score: a total score of 5-8 suggests a probable drug reaction, a score of 1-4 suggests a possible drug reaction, and a score of ≥9 and ≤0 implies a definite and doubtful drug reaction, respectively [19].

Question	Yes	No	Unknown	Score for sildenafil
Are there previous conclusive reports on this reaction?	+1	0	0	0
Did the adverse event occur after suspected drug administration?	+2	-1	0	+2
Did the adverse event improve on drug discontinuation or when a specific antagonist was administered?	+1	0	0	+1
Did the adverse event reappear when the drug was readministered?	+2	-1	0	0
Are there alternative causes that could, on their own, have caused the reaction?	-1	+2	0	+2
Did the reaction reappear when a placebo was given?	-1	+1	0	0
Was the drug detected in blood or other fluids in concentrations known to be toxic?	+1	0	0	0
Was the reaction more severe when the dose was increased or less severe when decreased?	+1	0	0	0
Did the patient have a similar reaction to the same or similar drugs in any previous exposure?	+1	0	0	0
Was the adverse event confirmed by any objective evidence?	+1	0	0	0
Total score	-	-	-	5

This suggested a probable drug reaction, further aiding our efforts in establishing a causal link. However, we were unable to obtain objective evidence of specific sildenafil-directed anti-drug antibodies, as the patient retrospectively disclosed consuming the drug in his second week of hospitalization. The serological detection of drug-dependent antibodies is critical to the diagnosis of DIIHA and involves testing the patient's serum or eluate prepared from the patient's RBCs with specific drug-coated RBCs and/or enzyme-treated RBCs tested in a solution containing a suitable concentration of the suspected drug [20]. This would require specialized equipment and some expertise in preparing the same, which was unavailable to us at the time and we acknowledge a limitation of this report.

## Conclusions

In this report, we wish to highlight the diagnostic dilemmas associated with a case of DIIHA. With a pathology closely mimicking other forms of immune hemolytic anemias, objective evidence of DIIHA is frequently missed, as it requires the demonstration of specific anti-drug antibodies, which necessitate specialized equipment and expertise, and is associated with lengthy turnaround times. Furthermore, in the event of a hemolytic crisis, blood transfusions and corticosteroid therapy, while critical to patient care, can profoundly hamper efforts in demonstrating anti-drug antibodies. While every suspected case of DIIHA warrants a systematic search and diagnostic workup, such diagnostic efforts must never be allowed to hinder patient care. A detailed drug history, at baseline and retrospectively when the working diagnosis is challenged internally, can frequently unearth unexpected results, as they did in our case.

This case highlights a possible association between unsupervised sildenafil use and the development of immune hemolytic anemia, complicated by AKI requiring renal replacement therapy. While the present report is limited by the lack of demonstration of drug-dependent antibodies, critical to diagnosing DIIHA, our utilization of subjective evidence (the Naranjo adverse drug reaction probability scale), supported by a significant drug history elicited retrospectively, allowed us to arrive at a diagnosis without impeding patient care. This is the first clinical report to implicate sildenafil in DIIHA, based on temporal association, clinical course, and exclusion of other known causes.

## References

[1] Garratty G, Arndt PA (2014). Drugs that have been shown to cause drug-induced immune hemolytic anemia or positive direct antiglobulin tests: some interesting findings since 2007. Immunohematology.

[2] Jäger U, Barcellini W, Broome CM (2020). Diagnosis and treatment of autoimmune hemolytic anemia in adults: recommendations from the First International Consensus Meeting. Blood Rev.

[3] Garratty G (2009). Drug-induced immune hemolytic anemia. Hematology Am Soc Hematol Educ Program.

[4] Hansen DL, Möller S, Andersen K, Gaist D, Frederiksen H (2020). Increasing incidence and prevalence of acquired hemolytic anemias in Denmark, 1980-2016. Clin Epidemiol.

[5] Maquet J, Lafaurie M, Walter O (2021). Epidemiology of autoimmune hemolytic anemia: a nationwide population-based study in France. Am J Hematol.

[6] Guha T, Kohad H, Bhar R (2016). Sildenafil citrate (viagra) reduces surface roughness of human erythrocytes: atomic-force-microscopic study. J Microsc Ultrastruct.

[7] Renard D, Rosselet A (2017). Drug-induced hemolytic anemia: pharmacological aspects. Transfus Clin Biol.

[8] Arndt PA (2014). Drug-induced immune hemolytic anemia: the last 30 years of changes. Immunohematology.

[9] Van Avondt K, Nur E, Zeerleder S (2019). Mechanisms of haemolysis-induced kidney injury. Nat Rev Nephrol.

[10] Westenfelder C, Gooch A (2022). Heme protein-induced acute kidney injury is caused by disruption of mitochondrial homeostasis in proximal tubular cells. Kidney360.

[11] Salama A (2009). Drug-induced immune hemolytic anemia. Expert Opin Drug Saf.

[12] Leicht HB, Weinig E, Mayer B, Viebahn J, Geier A, Rau M (2018). Ceftriaxone-induced hemolytic anemia with severe renal failure: a case report and review of literature. BMC Pharmacol Toxicol.

[13] Lemack B, Kupovics G, Kumar R (2025). Drug-induced immune hemolytic anemia following dapagliflozin administration: a case report. Cureus.

[14] McCullough AR (2002). Four-year review of sildenafil citrate. Rev Urol.

[15] Giuliano F, Jackson G, Montorsi F, Martin-Morales A, Raillard P (2010). Safety of sildenafil citrate: review of 67 double-blind placebo-controlled trials and the postmarketing safety database. Int J Clin Pract.

[16] Coons JC, Pogue K, Kolodziej AR, Hirsch GA, George MP (2019). Pulmonary arterial hypertension: a pharmacotherapeutic update. Curr Cardiol Rep.

[17] Khan AS, Sheikh Z, Khan S, Dwivedi R, Benjamin E (2011). Viagra deafness--sensorineural hearing loss and phosphodiesterase-5 inhibitors. Laryngoscope.

[18] Liu B, Meng L, Guan X, Gao L, Trabin J (2018). Reversible acute kidney injury associated with sildenafil overdose. Cureus.

[19] Naranjo CA, Busto U, Sellers EM (1981). A method for estimating the probability of adverse drug reactions. Clin Pharmacol Ther.

[20] Nguyen TN, Fihman V, Maenulein E, Vinatier I, Klaren JM (2020). Drug-induced immune hemolytic anemia investigation: comparison between tube test and microcolumn agglutination (gel test) for the detection of drug-dependent antibodies in the presence of soluble drug. Transfus Clin Biol.

